# Survival of primary total hip arthroplasty in rheumatoid arthritis patients: Findings in 1,661 arthroplasties in 1,395 patients from the Danish Hip Arthroplasty Registry

**DOI:** 10.3109/17453671003685418

**Published:** 2010-03-31

**Authors:** Christoffer Rud-Sørensen, Alma B Pedersen, Søren Paaske Johnsen, Anders Hammerich Riis, Søren Overgaard

**Affiliations:** ^1^Department of Orthopaedic Surgery, Odense University Hospital, and Clinical Institute, University of Southern DenmarkOdense; ^2^Department of Clinical Epidemiology, Aarhus University Hospital, AarhusDenmark

## Abstract

**Background and purpose:**

There has been a limited amount of research on survival of total hip arthroplasties (THAs) in rheumatoid arthritis (RA). We therefore performed a population-based, nationwide study to compare the survival of primary THAs in RA patients and in osteoarthritis (OA) patients. We also wanted to identify predictors of THA failure in RA patients.

**Methods:**

Using the Danish Hip Arthroplasty Registry, we identified 1,661 primary THAs in RA patients and 64,858 in OA patients, all of which were inserted between 1995 and 2008. The follow-up period was up to 14 years for both groups.

**Results:**

Regarding overall THA survival, the adjusted RR for RA patients compared to OA patients was 0.81 (95% CI: 0.65–1.01). We found no difference in survival of cups between primary THAs in RA and OA patients. In contrast, there was better overall survival of stems in RA patients than in OA patients, both regarding revision due to aseptic loosening (adjusted RR = 0.58; 95% CI: 0.34–0.99) and for any reason (adjusted RR = 0.63; 95% CI: 0.45–0.88). In RA patients, males had a higher risk of revision than females concerning aseptic loosening of the stem, any revision of the stem, and any revision of both components.

**Interpretation:**

The overall survival of primary THAs in RA patients is similar to THA survival in OA patients. Stem survival appeared to be better in RA patients, while survival of the total THA concept did not show any statistically significant differences between the two groups. In RA patients, males appear to have a greater risk of revision than females.

## Introduction

Next to osteoarthritis (OA), rheumatoid arthritis (RA) is the commonest reason for total hip arthroplasty (THA) (Annual Report, Danish Hip Arthroplasty Registry, 2009). Several characteristics of RA patients, including the destructive nature of the disease, poor bone stock, the use of cytostatics or glucocorticoids for treatment, and an increased level of comorbidity, may possibly contribute to a poorer THA survival in this group.

Research on survival of THAs in RA patients has been limited. Some (Unger et al. 1987, Snorrason et al. 1993, Lachiewicz 1997, Creighton et al. 1998, Jana et al. 2001, Tang and Chiu 2001) but not all authors (Kirk et al. 1993, Ranawat 1998, Furnes et al. 2001, Eskelinen et al. 2006) have reported a poor prognosis of THA in RA patients, but the findings have been inconsistent. Several design issues may have undermined the existing study results, including small study samples, lack of long-term follow-up, incomplete follow-up, and lack of control groups (e.g. OA patients). Thus, the prognosis of THA in RA patients remains somewhat uncertain.

The main aim of our study was to compare THA survival in RA and OA patients. In addition, we wanted to identify patient- and surgery-related predictors of revision in RA patients.

## Patients and methods

### Settings and design

In Denmark, the National Health Service provides tax-supported healthcare for all inhabitants, allowing free access to general practitioners and hospitals. At birth, all Danish citizens are assigned a unique 10-digit personal identification number, which is used in all public registries.

### Civil registration system

This registry has kept information (updated on a daily basis) on death, emigration, and change of address for all Danish national citizens since 1968.

### The Danish National Registry of patients

The National Registry of Patients was established in 1977, and keeps data concerning both admissions and discharges of patients, including up to 20 discharge diagnoses, and all surgical procedures performed in the Danish hospitals. The diagnoses are classified according to the Danish version of the International Classification of Diseases (eighth edition up to 1993 and tenth edition thereafter). From the registry, we collected comorbidity data on all THA patients since 1977 and constructed the Charlson comorbidity index (Charlson et al. 1987). This index, which was validated in 2003 (de Groot et al. 2003), includes 19 major disease categories including cardiovascular, cerebrovascular, chronic pulmonary, liver, renal and ulcerative diseases, diabetes, and solid and hematological tumors. Admissions from each category are weighted as 1, 2, 3, or 6 points, and the score is the sum of these weights. We classified all patients according to 3 levels of comorbidity: low-index (individuals with a score of 0 prior to the time of surgery), moderate (individuals with 1 or 2 points), and high-index (individuals with more than 2 points).

### The Danish Hip Arthroplasty Registry (DHR)

We based our study on information from the DHR (Annual Report, Danish Hip Arthroplasty Registry, 2009), which was established in January 1995. The main purpose of the DHR is to improve the quality of both primary and revision THA surgery in Denmark. All orthopedics departments, including private hospitals, in Denmark submit detailed pre- and peroperative data concerning THA surgeries to the DHR. These data have been recorded using a standardized form, which is filled in by the orthopedic surgeon immediately after surgery. The completeness and validity of the registry has already been evaluated (Pedersen et al. 2004) and afterwards the form became part of the daily routine in the registry settings.

Since then, the completeness of the DHR has been compared with the Danish National Registry of patients (which is assumed to be the gold standard for registration of all procedures in Denmark) every 3 months. Afterwards, the orthopedic departments receive a list with all the patients, who have not been registered in the DHR, and are thereafter responsible for registering those patients. The result of this routine process has been reflected in the annual completeness of the DHR of more than 90% for the last 10 years (Annual report, Danish Hip Arthroplasty Registry, 2009). Validation of several important variables included in the DHR with corrections based on the medical records at department level is further performed at least once a year.

Our study population consisted of 1,661 primary THAs in RA patients and 64.858 primary THAs in patients with OA. Altogether, 266 RA patients (16%) and 10,636 OA patients (16%) had bilateral THA, more than 90% of which were two-stage procedures. It has been shown previously that inclusion of dependent observations such as bilateral procedures on the same patients does not influence revision risk, even though the proportion of individuals undergoing bilateral surgery can be almost 20% (Robertsson and Ranstam 2003).

### Outcome

Time to revision of the THA was taken as the endpoint. Revision was defined as removal or exchange of either of the components or parts. Both aseptic loosening and any cause of revision were considered endpoints, and patients were followed until death, emigration, revision, or December 31, 2008.

### Statistics

The Cox regression model was used to calculate both unadjusted and adjusted hazard ratios for revision with 95% confidence interval (CI) for RA compared with OA patients. The hazard ratio was used as a measure of relative risk (RR). We adjusted for age (0–59 years (reference), 60–69 years, 70–79 years, and 80+ years), sex (female (reference)), comorbidity (Charlson score 0 (reference), 1–2, and 3+), duration of surgery (continuous variable), and type of fixation (uncemented (reference), and cemented prostheses). We compared distributions using chi-squared test. 2-sided p-values < 0.05 were considered to be statistically significant. To describe time to revision and absolute risk of revision, we constructed Kaplan-Meier plots to illustrate cumulative incidence of revision. The assumption of the Cox proportional hazards model was assessed graphically using log-log plots and found suitable.

Finally, we examined the association with time to revision for a broad range of patient- and surgery-related factors in RA patients in order to identify possible predictors of revision.

The statistical analyses were performed using SAS software version 9.2. The study was approved by the Danish Data Protection Agency (j. no. 2009-41-3644).

## Results

### Patient characteristics (Table 1)

Most RA patients were females younger than 60 years of age and had a higher comorbidity score before surgery than OA patients, while the distribution of cemented and uncemented prosthetic components appeared to be similar in the RA and OA groups. Median age at the time of primary surgery was 64 and 70 years in RA and OA patients, respectively. The median length of follow-up was 5.9 (25 to 75 percentile: 3.1–9.1) years for the RA group and 4.7 (25 to 75 percentile: 2.2–7.8) years for the OA group.

**Table 1.** Characteristics of THAs, patients with rheumatoid arthritis (RA), and patients with osteoarthritis (OA)

	RA patients		OA patients		p-value
	n	%	n	%	
Gender	Female	1203	72	36,498	56	–
	Male	458	28	28,360	44	< 0.001
Age	0–59 years	595	36	11,115	17	–
	60–69 years	544	33	21,306	33	–
	70–79 years	435	26	23,418	36	–
	> 80 years	87	5	9,019	14	< 0.001
Comorbidity						
	Low (0)	218	13	44,163	68	–
	Moderate (1–2)	1,256	76	17,010	26	–
	High (3+)	187	11	3,685	6	< 0.001
Cup	Cemented	783	47	28,077	43	–
	Uncemented	878	53	36,781	57	0.002
Stem	Cemented	1,215	73	44,360	68	–
	Uncemented	446	27	20,498	32	< 0.001
Total	-	1,661	100	64,858	100	

### Risk of revision for total hip arthroplasty (including cup and stem) (Table 2)

The risk of revision of the entire cup/stem concept appeared to be lower in RA patients than in OA patients (adjusted RR of revision for any reason = 0.81; CI: 0.65–1.01); however, the difference did not reach statistical significance. We found similar revision risks in RA and OA patients according to three time periods; thus, adjusted RR was 0.92 (CI: 0.69–1.22) for RA vs. OA patients operated in the period from 1995 through 1998, adjusted RR was 0.72 (CI: 0.47–1.10) for RA vs. OA patients operated in the period between 1999 and 2003, and adjusted RR was 0.57 (CI: 0.29–1.11) for RA vs. OA patients operated in the period among 2004 and 2008.

**Table 2.** Risk of revision in THA patients with rheumatoid arthritis (RA) compared to that in osteoarthritis (OA) patients. The p-values are connected to the adjusted RR

	Cause of revision	RA		OA		Crude RR (95% CI)	Adjusted RR**^a^** (95% CI)	p-value
		n	%	n	%			
Cup	Aseptic loosening	30	1.8	658	1.0	1.37 (0.95–1.98)	1.14 (0.77–1.68)	0.5
	Any cause	74	4.5	2,024	3.1	1.21 (0.96–1.53)	0.94 (0.74–1.20)	0.6
Stem	Aseptic loosening	15	0.9	752	1.2	0.61 (0.37–1.02)	0.54 (0.32–0.92)	0.02
	Any cause	48	2.9	2,082	3.2	0.77 (0.58–1.02)	0.62 (0.46–0.83)	< 0.01
Cup and stem	Any cause	89	5.4	2,979	4.6	1.00 (0.81–1.23)	0.81 (0.65–1.01)	0.07
**^a^**Relative risk of revision with 95% CI, mutually adjusted for age, gender, comorbidity, duration of surgery, and type of fixation.								

### Risk of cup revision (Table 2)

**Figure 1.**
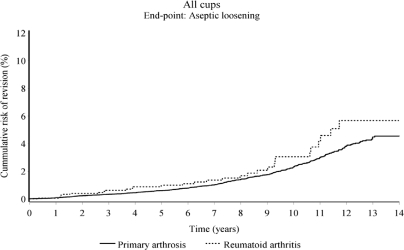
The cumulative risk of revision of cups in RA patients and OA patients for aseptic loosening.

The cumulative risk of revision of the cup due to aseptic loosening was slightly higher for RA patients than for OA patients during the entire study period, being 1.0% (CI: 0.6–1.7) and 0.6 (CI: 0.5–0.7) in RA and OA patients at 5 years follow-up, and reaching 5.7% (CI: 3.7–8.8) in RA patients and 4.6% (CI: 4.0–5.1) in OA patients after 14 years of follow-up (Figure 1). Although it may appear that the revision risk was higher in RA patients, after adjusting for possible confounders we found no statistically significant difference in the risk of revision due to aseptic loosening of the cup in RA patients compared to OA patients (adjusted RR = 1.14; CI: 0.77–1.68).

**Figure 2.**
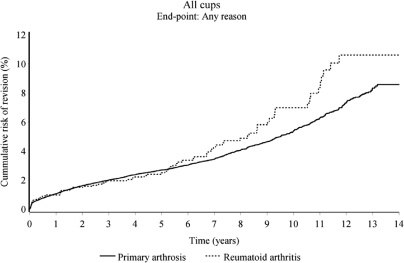
The cumulative risk of revision of cups in RA patients and OA patients for any reason.

We did not find any statistically significant difference in the risk of revision for any reason (adjusted RR = 0.94; CI: 0.74–1.20) between cups in RA patients compared to OA patients. After 5 years, the cumulative risk of revision for RA and OA patients was 2.5% (CI: 1.8–3.5) and 2.7% (CI: 2.6–2.8), respectively, and after 14 years the risk had increased to 11% (CI: 8.0–14) for the RA group and 8.6% (CI: 7.9–9.3) for the OA group (Figure 2).

### Risk of stem revision (Table 2)

**Figure 3.**
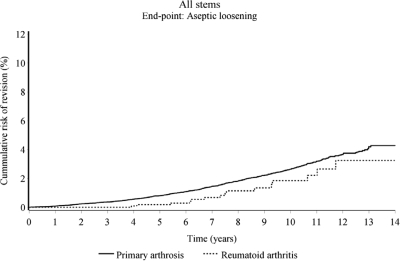
The cumulative risk of revision of stems in RA patients and OA patients for aseptic loosening.

**Figure 4.**
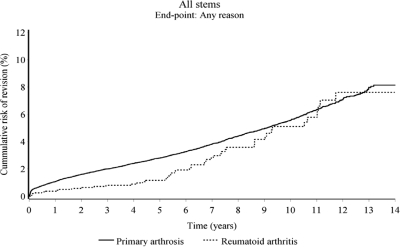
The cumulative risk of revision of stems in RA patients and OA patients for any reason.

The cumulative risk of stem revision due to aseptic loosening was 0.2% (CI: 0–0.7) in RA patients and 0.8% (CI: 0.7–0.9) in OA patients at 5 years, and after 14 years the corresponding figures were 3.2% (CI: 1.8–5.9) and 4.3% (CI: 3.8–4.8). Nevertheless, at the 5-year follow-up the cumulative risk of revision of the stem for any reason was 1.2% (CI: 0.7–1.9) for RA patients and 2.8% (CI: 2.7–2.9) for OA patients, reaching 7.6% (CI: 5.4–11) for RA patients and 8.1% (CI: 7.5–8.8) for OA patients (Figures 3 and 4). After adjusting for possible confounding factors, we found statistically significantly lower risk of revision in stems in RA patient than in OA patients regarding both aseptic loosening (adjusted RR = 0.54; CI: 0.32–0.92) and revision for any reason (adjusted RR = 0.62; CI: 0.46–0.83).

### RA subgroup

We found an increased risk of stem revision in males than in females concerning both aseptic loosening (adjusted RR = 8; CI: 2.5–25) and revision for any reason (adjusted RR = 2.3; CI: 1.3–4.1). Furthermore, we found an increased risk of revision in males than in females regarding the total stem/cup concept and any cause of revision (adjusted RR = 1.7; CI: 1.1–2.7). Otherwise, we found no difference in the risk of revision due to aseptic loosening or any revision according to age, Charlson comorbidity index, duration of surgery, or fixation technique.

## Discussion

In this large nationwide population-based follow-up study, we found that the risk of revision following THA in RA patients was lower or similar to that in OA patients. Stems had a better survival in RA patients than in OA patients.

### Strengths and limitations of our study

The main strengths of our study were its population-based prospective design with almost complete follow-up, and the large sample size. In addition, a previous study (Pedersen et al. 2004) reported a high validity of data in the Danish Hip Arthroplasty Registry, with 94% completeness of procedures registered and a positive predictive value of between 84% and 100% for hip diagnoses for primary total hip replacement. In addition, our study was based on data collected independently of its objective. Completeness of registration of revisions in the DHR is approximately 90%. The lack of registration of revisions in the DHR (approximately 10%) is most likely non-differential, i.e. independent of hip diagnosis for THA (Pedersen et al. 2004). Furthermore, we adjusted for a wide range of possible confounders in the statistical analyses, including age, sex, comorbidity history, duration of surgery, and fixation technique. Previous studies did not include comorbidity data in the analyses as a confounder, although this is a well-established prognostic factor for implant survival (Johnsen et al. 2006).

The study also has some limitations, however. First of all, the absolute number of revisions was moderate to low, which is reflected in the statistical precision of the risk estimates. Even though the follow-up ranged from 0 to 14 years, the relative short-term median follow-up of 5.9 and 4.7 years for RA and OA patients, respectively, precluded us from making conclusions on long-term results for THAs in RA patients.

Although we adjusted for some confounders, we were not able to adjust for other possible confounders such as tobacco use, alcohol use, social status, and use of drugs, which might affect the prognosis of the THA if they were related to primary hip diagnosis. Finally, the DHR does not collect data on patient-related information regarding quality of life, especially concerning pain and level of physical activity before and after the surgery, which may have had an effect on implant survival as well.

### RA vs. OA

The cumulative risk of revision of the cups in RA patients found in our study is in accordance with the results from the Finnish register-based study (Eskelinen et al. 2006). However, comparison of the results is challenging because the Finnish study population is younger and the implant concepts used in RA patients are not quite the same as those used in Denmark. 2 other clinical studies (Ahnfelt et al. 1990; Furnes et al. 2001) have reported an increased risk of revision of cups in RA patients, attributing the poor results partly to poor bone stock (Snorrason et al. 1993). 2 radiological studies have also confirmed these poor results with cups in RA patients (Carlsson et al. 1986; Kobayashi et al. 1994), but these studies were only based on Charnley prostheses.

Several authors have reported good results concerning the survival of stems in RA patients (Jana et al. 2001, Keisu et al. 2001, Effenberger et al. 2002, Eskelinen et al. 2006), but most of these studies did not include a control group. We do not know for certain what causes RA stems to last longer than OA stems, but perhaps it can be explained by a lower level of physical activity in RA patients.

Furnes et al. (2001) found no difference in the 10-year survival rate of total implants between RA patients and OA patients, which is in accordance with our results. Similar results were reported in a recent paper from the DHR (Johnsen et al. 2006). However, since the absolute difference in cumulative revision rate estimates between RA and OA patients was small during the median follow up time of approximately 5–6 years, the clinical implication of our findings is unclear.

### RA subanalysis

Several earlier studies on RA patients have found a lower risk of revision in older patients than in younger patients (Lehtimäki et al. 1999, Furnes et al. 2001, Eskelinen et al. 2006). We could not confirm these previous findings. However, we did find a higher risk of revision in men than in women concerning both the stem and the THA as a whole. This difference between males and females was also reported by Eskelinen et al. (2006) and further confirmed by Lehtimäki et al. (1999), Furnes et al. (2001), and Johnsen et al. (2006) for THA patients in general, including RA patients. The mechanism underlying the association between revision risk and male sex has not been well established. However, it appears likely that occupational lifting in men (Coggon et al. 2006), women’s concerns about surgical complications and surgical implications for their family and therefore less willingness to discuss the possibility of surgery with their physician (Hawker et al. 2000), and a higher referral rate from primary care physicians to any kind of specialist for male patients and thus a higher possibility of receiving surgery (Franks and Clancy 1997), may play role in this association.

A previous study from the DHR has shown that comorbidity is an important predictor regarding the survival of primary THAs (Johnsen et al. 2006). Thus, a high Charlson comorbidity index was associated with an increased risk of revision of all THAs, including RA and OA. We were unable to confirm these results for our RA patients, which could partly be due to the low number of RA patients with no comorbidity in our study. Even so, the issue of confounding by indication may blur our estimated risk, as RA patients with a moderate or high level of comorbidity may have a lower likelihood of being offered revision of a malfunctioning prosthesis compared to OA patients due to the anticipated higher operative risk.

In several studies, aseptic loosening of cemented cups has been found to be disturbingly high (Cracchiolo et al. 1992, Lachiewicz 1997, Unger et al. 1997, Creighton et al. 1998, Ranawat 1998, van der Lugt et al. 2003). We did not find such poor results regarding the cemented cups, either due to aseptic loosening or for any cause of revision. These findings are in accordance with 2 other large registry studies (Furnes et al. 2001, Eskelinen et al. 2006). Eskelinen et al. (2006) found promising results for cemented polyethylene cups, with a 10-year survival of more than 90%, and based on these results they recommended this type of prosthesis as an alternative in younger patients. Furnes et al. (2001) did not find any significantly worse results either, concerning this issue. The good results with cemented cups (compared to uncemented cups) reported lately may have partly contributed to several substantial improvements in modern cementing techniques during the past 10–15 years.

The good survival of cemented and uncemented stems in RA patients found in our study is in accordance with the results of several larger studies (Furnes et al. 2001, Jana et al. 2001, Eskelinen et al. 2006). These authors all described good results concerning uncemented porous-coated stems, while Cracchiolo et al. (1992) described good results with several different uncemented prostheses.

In conclusion, the overall survival of primary THAs in RA patients is comparable if not better than the survival found in OA patients. We found a significantly lower risk of stem revision in RA patients than in OA patients, and furthermore we found that male sex was a risk factor for stem revision in RA patients.
